# Identification of COVID-19 Spreaders Using Multiplex Networks Approach

**DOI:** 10.1109/ACCESS.2020.3007726

**Published:** 2020-07-07

**Authors:** Edwin Montes-Orozco, Roman-Anselmo Mora-Gutiérrez, Sergio-Gerardo De-Los-Cobos-Silva, Eric-Alfredo Rincón-García, Gilberto-Sinuhe Torres-Cockrell, Jorge Juárez-Gómez, Bibiana Obregón-Quintana, Pedro Lara-Velázquez, Miguel-ángel Gutierrez-Andrade

**Affiliations:** 1 Posgrado en Ciencias y Tecnologías de la InformaciónUniversidad Autónoma Metropolitana Iztapalapa 27788 Mexico City09340Mexico; 2 Departamento de SistemasUniversidad Autónoma Metropolitana Azcapotzalco 27787 Mexico City02200Mexico; 3 Departamento de Ingeniería EléctricaUniversidad Autónoma Metropolitana Iztapalapa 27788 Mexico City09340Mexico; 4 Departamento de QuímicaUniversidad Autónoma Metropolitana Iztapalapa 27788 Mexico City09340Mexico; 5 Facultad de CienciasUniversidad Nacional Autónoma de México 7180 Mexico City04510Mexico

**Keywords:** Complex networks, complex systems, COVID-19, multiplex networks, optimization, social networks

## Abstract

In this work, we present a methodology to identify COVID-19 spreaders using the analysis of the relationship between socio-cultural and economic characteristics with the number of infections and deaths caused by the COVID-19 virus in different countries. For this, we analyze the information of each country using the complex networks approach, specifically by analyzing the spreaders countries based on the separator set in 5-layer multiplex networks. The results show that, we obtain a classification of the countries based on their numerical values in socioeconomics, population, Gross Domestic Product (GDP), health and air connections; where, in the spreader set there are those countries that have high, medium or low values in the different characteristics; however, the aspect that all the countries belonging to the separator set share is a high value in air connections.

## I. Introduction

In the current year (2020), the world has faced a disease caused by SARS-CoV-2, known as COVID-19. This virus began to spread in Wuhan China on December 31, 2019 [1] and, because the virus has spread rapidly throughout the world, it has been determined as a pandemic by the World Health Organization (WHO) in January 2020 [2].

COVID-19 is classified as a virus composed of single-stranded RNA strands, and the symptoms of the disease caused by COVID-19 are headache, dry cough, malaise, fever, and respiratory failure [3]. However, cases of asymptomatic people against the virus have been identified, which implies a real challenge for health institutions.

As of March 31, 2020, the United States has become the epicenter of the pandemic, followed by Italy, Spain, China, and Germany with 186,265, 105,792, 95,923, 82,278, and 71,690 confirmed cases, respectively [4]. These five countries represent 63.4% of the total confirmed cases worldwide. The recovery percentages of this new pandemic are led by China, Spain, Germany, Italy, and Iran with 42.8, 10.8, 9.1, 8.8, and 8.2% of cases. It is essential to mention that in Europe and America, the spread of COVID-19 throughout the world can be attributed to differences in health infrastructure, air travel, human development, and other socio-cultural and economic factors [5]–[7].

Therefore, in this work, we analyze the effect of socio-cultural and economic factors, air travels, human development on both spreading and growth of COVID-19 in each country, using the Vertex Separator Problem (VSP) [8] in multiplex complex networks. Here, it is essential to mention that the data for each topic was obtained from the websites of the European Union (EU) [9], the World Health Organization (WHO) [4], the World Bank (WB) [10], the International Monetary Foundation (IMF) [11] and Transparency International (TI) [12] until May 15, 2020.

On the other hand, a complex network is a network with non-trivial topological characteristics that do not occur in simple networks, such as degree distributions, hierarchical structures, community structures, and high local cohesiveness (measured through the clustering coefficients) [13].

The identification and quantification of influential nodes in complex networks is an essential activity in several application fields, such as the spread and control of diseases [14], the identification of the most influential members of a criminal group [15], to know how impactful are the academic publications [16], to predict future relationships [17]–[19], among others [20], [21].

Currently, there are several measures to identify influential or spreaders nodes in complex networks, where the most classic and important are:

• Closeness Centrality (CC) [22]: Classifies the importance based on the inverse sum of the shortest distances to all other nodes from a central node, measuring the global structure.
• Degree centrality (DC) [23]: Classifies each node based on its degree; therefore, the more connections a particular node has, the more important it is considered.
• Betweenies Centrality (BC) [22]: Classifies the nodes by the number of the shortest paths between any other pair of nodes that cross through it.

In recent years, the study of complex networks and specifically of multilayer networks has been emphasized; this is thanks to the fact that most real systems have structures with multiple types of links or interactions between nodes; for example: Multimodal transport systems, biological systems, social networks and numerous modes of communication [24].

Multiplex networks are a particular class of multilayer networks, which were introduced to better model complex real-world systems [25], [26]. The main characteristic of multiplex networks is that all the nodes in each layer are replicated in other layers, and there is a direct link between each replica node to denote the relationship.

Formally, let $\text {GP}=(\mathcal {G_{\alpha }}, \mathcal {C})\,\,\forall {}\alpha {}\in {} \{1,\ldots M\}$, be a multiplex network where:

• $\mathcal {G_{\alpha }} = (X_{\alpha }, E_{\alpha })$, is a monoplex network called layer $\alpha $, where $X_{\alpha }$ and $E_{\alpha }$ are the set of nodes^1^ and the links in layer $\alpha $, respectively.
• $\mathcal {C}=\{E_{\alpha \beta } \subseteq X_{\alpha } \times X_{\beta }; \text {$\alpha $, $\beta ~\in ~\{1,\ldots,M\}$}, \alpha \neq \beta \}$; is the set of interconnections between nodes in different layers. The elements of $\mathcal {C}$ are called cross layers and the elements of each $E_{\alpha }$ are called intralayer connections of GP.

^1^It is important to mention that in multiplex networks, each node belongs to all the $M$ layers.

The present work is organized as follows: In Related work, we show the main works indicated in the specific literature from previous years related to COVID-19, complex networks, spreaders nodes, and robustness in multiplex networks. In Materials and methods, we describe the main characteristics and the modeling process for monoplex and multiplex networks. In Results, we show the study of the numerical results. In Limitations of the study and discussion, we present the main characteristics and limitations of the work and finally, in Conclusions, we describe the summary of the work.

## II. Related Work

As mentioned above, the world is facing a pandemic caused by COVID-19 and, the analysis developed in this work shows that there is different behavior in the development of the disease in different countries. The identification of most spreader nodes in complex systems has led to the development of different methodologies; which are based on the calculation of the structural information of the network or the analogies of statistical physics or mathematical models.

At this point, it is essential to mention that we use the multiplex network approach since there are at least five types of complex systems to analyze (for more information, see Materials and Methods); therefore, we present the main works denoted in the specific literature related to multiplex networks, robustness, spreaders nodes, and COVID-19.

For the methodologies to identify spreaders nodes in networks based on structural information, the most representative works are:

• Zhao *et al.* [27], present an index to calculate the influence of a node based on the number of communities to which it belongs.
• Berahmand *et al.* [28], propose a local approach based on the detection and expansion of central nodes. The proposed algorithm can detect all the communities of the graph in a network using local information and identify several functions of the nodes.
• Berahmand *et al.* [29], propose a new measure of semi-local centrality that can assign higher ranges or structural holes as better diffusers in the network; therefore, the proposed centrality avoids the selection of separators that are very close to each other.
• Berahmand *et al.* [30], demonstrate that, in data sets with a rich-club, it is better to use degree centrality to find influential nodes because it has linear time complexity and uses local information.
• Berahmand *et al.* [31], propose a new local classification measure to identify the influence of a node, using the propagation capacity of the nodes based on their essential location parameters, such as the degree of the node, the degree of its neighbors, the standard links between a node and its neighbors and the inverse clustering coefficient.
• Wang *et al.* [32], study the identification of influential spreaders in complex networks based on several centrality indices.
• His *et al.* [33] and Han *et al.* [34] propose some node classification algorithms based on the identification of structural holes. A structural hole is known as the phenomenon that occurs when a node connected to multiple local bridges (multiple communities) is removed, and space is produced.
• Li *et al.* [35], present a classified neighbor algorithm to quantify the nodal propagation capacity. The results show that the proposed algorithm can effectively control the outbreak of epidemics in many real-world systems.
• Wang *et al.* [36], propose a measure of influence to quantify the propagation capacity of nodes in complex networks.
• Yan *et al.* [37], propose a method that takes into account several aspects of node properties, including local topological characteristics, central location, propagation characteristics, and ownership of neighboring nodes.

Most of these strategies are based on the global characteristics of the networks, such as: PageRank [38], LeaderRank [39], Excentricity Centrality [40], ClusterRank [41], K-shell [42], Eigenvector Centrality [43] and Katz Centrality [44].

For statistical physics-based methods, most papers use the percolation model [45]–[47], which is known as the time when the giant component (GC) is formed; for example:

• Radicci *et al.* [48], present a methodology based on the filtration of links, finding the relationships that make a node more influential or spreader.
• Fei *et al.* [49], present a methodology based on the inverse square law, where the sum of the attraction between any pair of nodes is used to classify the influence of each node.
• Marone and Makse [46], present a model of immunization of a network against epidemics and argue that the most influential or spreader nodes can be mapped in the optimal filtration.
  On the other hand, several optimization models have been presented, where the most representative works are:
• Cheng *et al.* [50], propose a methodology based on the Influence Maximization Problem (IMP) [51], which identifies a set of nodes that maximizes the influence effect on the network.

Finally, there are some optimization methods to solve the network percolation problem [45], [52], and recently, Montes-Orozco *et al.* [53] proposed a methodology based on inverse percolation that causes a rupture of the GC in monoplex and multiplex networks.

The main advantage of optimization models is that they can guarantee the optimal solution to the problem. However, some methodologies depend on the topology of the network.

Furthermore, if the nodes are removed randomly, the effect on the transmission is very slight; but, if the deleted nodes are chosen carefully, they can cause the system to colapse. Therefore, finding the elements that are important to network connectivity is of great importance.

Specifically for COVID-19, several mathematical and statistical models have been used in order to predict the behavior of the pandemic and even to evaluate its economic costs. Yuan *et al.* [54], present a model denoted as iSEIR to chart the pandemic path in Wuhan China, and thus they could precede the date of maximum contagion. Messina *et al.* [55], developed a network-based model to define the molecular aspects of pathogenic phenotypes in coronavirus infections. Liang *et al.* [56], developed a mathematical model to predict the propagation rates of three cases of pneumonia: COVID-19, SARS, and MERS.

On the other hand, Chin *et al.* [57], propose a methodology to identify spatial superspreaders with daily passenger data from public transport in Singapore. Chatterjee *et al.* [58], developed a stochastic mathematical model to predict the number of infections in India. Cao *et al.* [59], present an analysis based on several mathematical models to assess the prevention actions that were carried out in Wuhan, and they include that the actions had a crucial effect on the spread of the pandemic.

Finally, Bragazzi *et al.* [60], and Pham *et al.* [61], discuss extensively computational techniques and information technologies, artificial intelligence, and Big Data can help manage the enormous amount of data derived from the present pandemic.

## III. Materials and Methods

In this section, we present the way of model the multiplex networks, and the methodology to analyze and identify the most spreader countries of COVID-19. This study is divided into four phases, which are:

1) Data collection: In this phase, we build the data set through a statistical analysis applied to the information obtained from the IMF, WB, WHO and IT.
2) Construction of networks: In this phase, based on the similarity of the characteristics for each country, we model the monoplex and multiplex networks.
3) Analysis of spreader nodes: For the modeled networks, we use an adaptation of the VSP to identify spreaders countries in multiplex networks.
4) Analysis of results: In this phase, we show the study of the countries that are classified as spreaders, which cause the rupture of the multiplex networks.

### A. Materials

As mentioned above, to model the networks used in this work, we use the information about COVID-19 available on the websites of the European Union (EU) and World Health Organization (WHO); while for the socio-cultural and economic data we use the information available on the websites of the World Bank (WB), the International Monetary Foundation (IMF) and Transparency International (TI).

The information of COVID-19 (infections and deaths), includes the period from 12/31/19 to 05/15/20 and the indicators used to define the socio-cultural-economic characteristics, are: projected real Gross Domestic Product (GDP) (2020), projected consumer prices (2020), special drawing rights (millions), quota (millions), human development index (HDI) (2009-2018), corruption perception index (2018), Gross national income (GNI) per capita, GNI per capita rank minus HDI rank, country population (population/$km^{2}$), real population density (hectares by person), Gini Coefficient, current health expenditure (% of GDP 2000-2016) and air travels (2019-2020).

Specifically, in this work, we modeled two 5-layer multiplex networks; where each layer represents the relationship between each pair of countries $\upsilon $ as follows:

• Layer 1 (L1), for Human Development Index and its components.
• Layer 2 (L2), for Human Development Index Trends, 1990-2018.
• Layer 3 (L3), for the Inequality-adjusted Human Development Index.
• Layer 4 (L4), for the air travels (inbound and outbound) between each country.
• Layer 5 (L5), L5.1 for the number of infections and deaths network, and L5.2 for the GDP and health network.

These relationships are given by the number of characteristics in which the countries are similar, and their quantification is obtained as follows:

• The Mahalanobis distance between each pair of countries is calculated.
• The median of the Mahalanobis distances is calculated.
• For each pair of countries with a distance less than the median, a link is added.

Here, it is essential to emphasize that, although GDP is frequently used to compare international economies or the air travels are used to analyze the population movement; the idea of modeling five different types of layers is to be able to carry out a classification based on a multi-criteria analysis that allows a stable assessment of the different elements included, thus streamlining the decision-making process.

**FIGURE 1.**
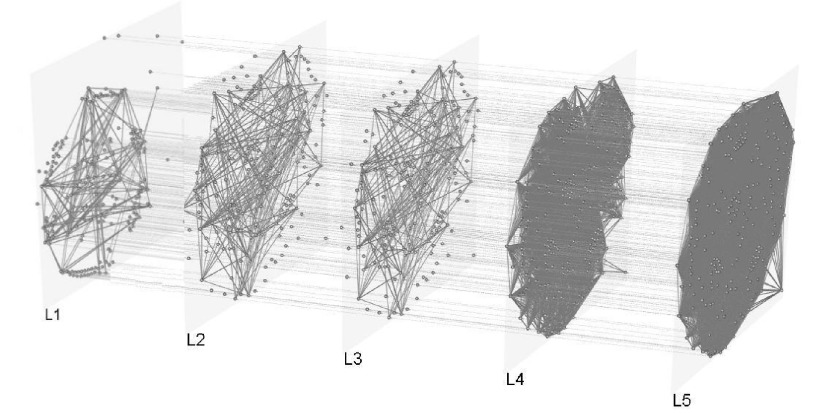
Graph of the infection multiplex network.

**FIGURE 2.**
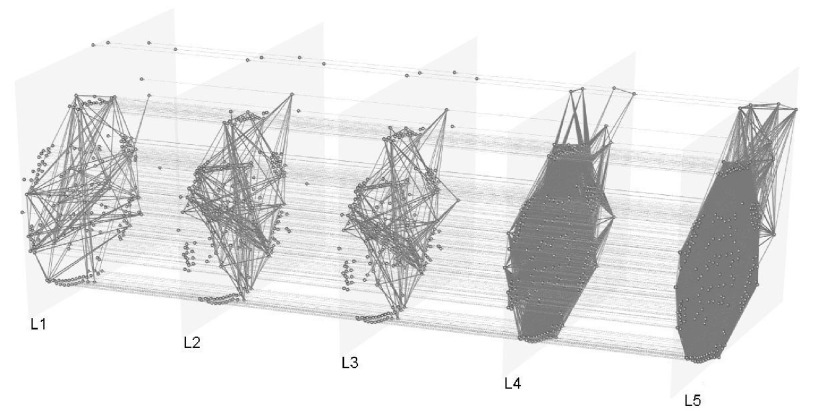
Graph of the GDP multiplex network.

Now, we present Figures 1 and 2, that show graphically the two 5-layer multiplex networks, denoted as infection and GDP, respectively.

In this study, each node represents a country, which belongs to all the layers of the multiplex network, where each interlayer link indicates that the two countries are similar in most of the characteristics analyzed, and the intralayer links indicate the identity relationship (main characteristic of multiplex networks).

Then, with the modeled networks, using the approach of VSP in multiplex networks, we develop an analysis about the influence of the countries and the propagation of COVID-19 using the information of the relationship of the main socio-cultural and economic characteristics, the number of flights, as well as the number of infections and deaths caused by COVID-19.

### B. Methods

In this work, the VSP is used to identify the spreaders countries. The approach of VSP is based on the robustness in networks finding the nodes that cause the rupture of the GC. In the case of multiplex networks, the GC is denoted as the Mutually Connected Giant Component (MCGC) defined in [62]:

Each node $i$ is in the MCGC if it has at least one neighbor $j$ that belongs to the MCGC and if all its replica nodes in each interdependent network are also in the MCGC.

From this definition, Bianconi *et al.* [62] deduce that if a node $i$ in a particular layer of a multiplex network is in an MCGC, then all its replica nodes in all layers are in the MCGC.

On the other hand, the VSP [53] consists of finding a minimum set of C nodes that, when their links are removed from the network, produce a disconnection from the multiplex network into at least two connected components (A, B), such that $|A|$ and $| B|$ are maximized. Then, the adaptation of VSP can be summarized as:

• Instance: A MCGC of a multiplex network $\text {GP}=(\mathcal {G_{\alpha }}, \mathcal {C})$.
• Problem: Find a partition of nodes belonging to the MCGC of GP that results into three disjoint sets $A$, $B$ and $C$, $A$ and $B$ nonempty, such that:

There are no interlayer or intralayer links between the elements belonging to each set $A$, $B$ or $C$.

$|A| \text {and }|B|$ are maximized.

$|C|$ is minimized.

It is essential to mention that we can quantify the robustness and the number of spreaders in the networks by analyzing the percentage of nodes that belong to the separator set $C$ (set of spreaders). For example, a high percentage (more than 70%) of nodes in $C$ indicates that the network is robust (there are a high number of spreader countries). In contrast, a low percentage (less than 30%) indicates that the network is not very robust (there are a low number of spreader countries).

Because the original VSP is considered as NP-Hard [8], [63], the adaptation from multiplex networks, is NP-Hard too [64], [65]; therefore, we can solve the adaptation of VSP using a heuristic method.

In this work, we used a simulated annealing algorithm (SA) [66] developed in C language. SA is a technique based on local search, and it is necessary to establish the structure of neighborhoods. In the development of this work, two neighborhoods were used: Hamming distance 1^2^ and Hamming distance 2. Then, the first type of neighborhood is added or deleted one node of the current solution with the following steps:

1) A random number between 0 and 1 is generated.
2) If random ≤ 0.5, one node is removed, otherwise one node is added.
3) To remove an element, it is chosen randomly, giving the same probability to each element that belongs to the current solution.
4) To add an element, it is chosen randomly, giving the same probability to each element that not belongs to the current solution.

^2^Hamming distance is defined as the number of elements that have to be changed to transform a solution into another valid solution.

For the other neighborhood, two nodes are removed, or two nodes are added, and if it is the case that two nodes can not be removed or added, one is deleted, and a different one is added. With this, the distance of Hamming 2 is maintained.

By last, the algorithm finished when the best solution it is not updated when 50 temperature updates have elapsed or $T_{k}=T_{f}$.

SA requires 4 control parameters and, in order to obtain a good performance it is important to find the effective parameter settings for the technique. Then, using the Differential Evolution algorithm (DE) [67]; we obtain the following values (for more information, see [68]):

• Initial temperature $T_{i} = 1500$.
• Final temperature $T_{f} = 0.001$.
• Cooling program $T_{k} \gamma $, with $\gamma = 0.95$.
• Times that a new neighbor is generated $L_{k}= 20$.

## IV. Results

In this section, we show the results and discussion on the main structural metrics of the modeled networks and the countries that cause the spread of COVID-19.

### A. Structural Metrics

In order to facilitate the understanding of the results of the networks structural metrics, we briefly introduce some fundamental concepts:

• Clustering coefficient [69]. It quantifies how much a node is interconnected with its neighbors; where, two nodes are neighbors if exist a link that join them, and for non-directed graphs, it is calculated in the following way:
  \begin{equation*} C_{i}=\frac {2|\{e_{ab}\}|}{k_{i}(k_{i}-1)}:\quad v_{a},v_{b} \in N_{i}, ~ e_{ab}\in E\end{equation*}
  where, $k_{i}$ is the degree of the node $v_{i}$; $v_{a}$ and $v_{b}$ belong to the neighborhood for the node $v_{i}$ ($N_{i}=k_{i}$) and; $e_{ab}$ is a subset of the total number of links ($E$) that connect any pair of nodes $v_{a}$, $v_{b}$.
  It is essential to mention that, the higher the number of triangles, the greater is the clustering coefficient value.
• Average degree [70]. The degree of a node is the number of links connected to it. Then, the average degree is calculated with the degrees of all the nodes of the network.
• Diameter of the network [71]. It is given by the longest route of all the shortest paths between any pair of nodes.
• Average path length [72]. Defines the average number of steps that must be traveled through the shortest path for all possible pairs of nodes.

In Table 1, we present the numerical values for the average, variance and the low and high limits for each structural metric and the closeness and betweenies centralities with 95% level of certainty (for each layer).

**TABLE 1:** Structural Metrics of Layers

ID		Degree	Clust. C.	Triangles	Closeness C.	Betweenies C.	Diam.	Path L.
L1	Av.	35.630	0.933	822.361	0.812	0.832	2	1.042
	Var.	421.636	0.012	335328.341	0.042	0.047		
	Low L.	34.178	0.921	741.032	0.742	0.815		
	High L.	40.083	0.945	921.225	0.842	0.845		
L2	Av.	38.761	0.215	823.263	0.815	0.842	3	1.099
	Var.	422.462	0.092	383342.402	0.0624	0.049		
	Low L.	33.712	0.159	729.631	0.763	0.832		
	High L.	43.826	0.271	916.831	0.831	0.853		
L3	Av.	37.405	0.946	837.774	0.824	0.856	4	1.322
	Var.	423.726	0.015	395358.351	0.064	0.051		
	Low L.	34.515	0.929	749.520	0.789	0.824		
	High L.	40.294	0.964	926.028	0.860	0.888		
L4	Av.	190.342	0.992	20129.969	0.971	0.985	2	1.056
	Var.	4.841	0.001	4842.545	0.001	0.002		
	Low L.	186.893	0.989	20120.201	0.970	0.984		
	High L.	193.257	0.994	20139.736	0.973	0.986		
L5.1	Av.	3.437	0.191	5.964	0.203	0.203	1	1
	Var.	10.066	0.145	217.914	0.029	0.029		
	Low L.	2.991	0.138	3.892	0.146	0.146		
	High L.	3.882	0.245	8.036	0.260	0.260		
L5.2	Av.	54.869	0.654	1239.767	0.549	0.634	3	1.86
	Var.	883.359	0.022	883668.239	0.020	0.028		
	Low L.	50.697	0.633	1107.825	0.529	0.610		
	High L.	59.040	0.675	1371.709	0.569	0.657		

Based on the information presented in Table 1, we can see that L1 to L4 and L5.2, have characteristics of the small-world model [73], while the layer 5.1 has characteristics of the scale-free model [74].

For example, L3 has a clustering coefficient of 0.952 and an average path length of 1.32, while L5.1 has 0.191 and 1, respectively. On the other hand, for closeness and betweenies centralities, L1 to L4 and L5.2 have high values, while L5.1 has low values.

### B. Identification of Spreaders of COVID-19

In Table 2, we present the numerical values obtained by applying the adaptation of VSP to the MCGC of Infections and GDP networks. In the first column, we show the identifier for each network; in the second column, we present the values for $|C|$, and; in the third column, we show the values for $|A|$ and $|B|$.

**TABLE 2:** Cardinality of Components $A$, $B$ and $C$

Network	|C|	|A| and |B|	Isolated components
Infection	108	42 and 43	3
GDP and health	111	36, 26 and 11	12

It is essential to mention that, in both networks, the MCGC is the total of the nodes (195 countries). On the other hand, we must remember that if $|C|$ has a value higher than 70% of the total nodes of the MCGC, it is considered robust (there are many spreader nodes). On the other hand, when $|C|$ has a value lower than 30%, the network is not very robust (there are few spreader nodes).

According to the results shown in Table 2, we can verify that applying the adaptation of VSP; we can find the set of nodes that cause the rupture of the MCGC for the 5-layer multiplex networks, since the sum of $|A|$, $|B|$, $|C|$ and the isolated components (nodes without connections to other nodes) is equivalent to the total number of nodes that belong to the MCGC.

Furthermore, we can assure that the networks are not robust and that there are many spreader nodes; therefore, we can deduce that the elimination of the links of the countries that are in the separator set, the pandemic caused by COVID-19 can be controlled, since in both networks $|C|$ is 54% and 56% of the total nodes belonging to the MCGC, respectively.

Then, in order to analyze the countries that cause the rupture of the MCGC in the networks, we perform an analysis of the types of countries that cause the spread of COVID-19.

**FIGURE 3.**
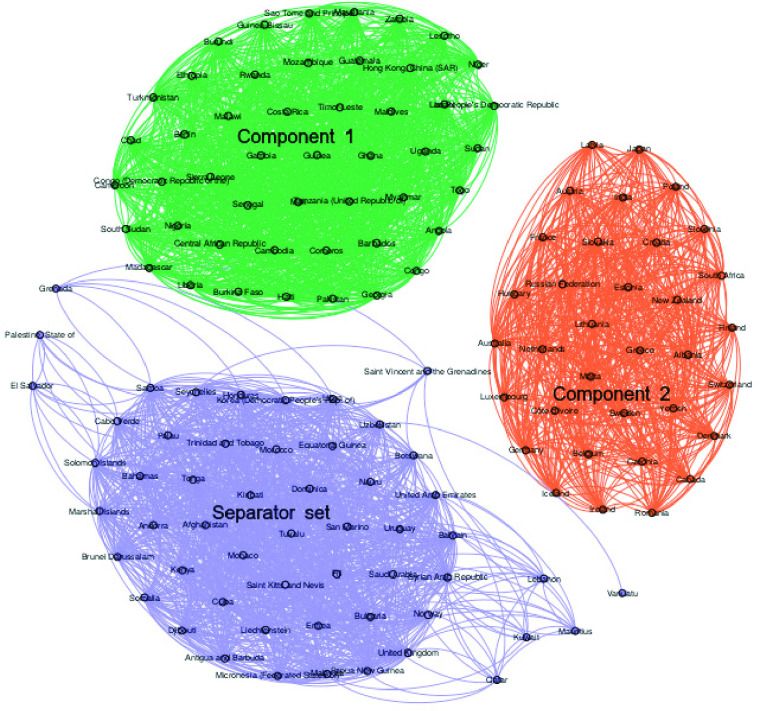
Graph of the sets of countries after the rupture of the MCGC (infection network).

Now, we present Table 3 and Figure 3), which show the countries that belong to each set after the rupture of the MCGC for the infection network.

**TABLE 3:** Sets of Countries After the Rupture of the MCGC (Infection Network)

Infection network	
Separator set	Norway, Ireland, Hong Kong, Australia, Sweden, Singapore, Netherlands, Denmark, United Kingdom, Liechestein, Austria, Luxembourg, India, Israel, France, Lithuania, Andorra, Qatar, Chile, Croatia, Argentina, Kazahstan, Romania, Kuwait, Bahamas, Malaysia, Serbia, Trinidad and Tobago, Iran, Mauritius, Panama, Georgia, Sri Lanka, Cuba, Saint Kitts and Nevis, Antigua y Barbuda, Mexico, Armenia, Algeria, Nicaragua, North Macedonia, Peru, Tunisia, Mongolia, Lebanon, Jamaica, Venezuela, Suriname, Belize, Maldives, Tonga, Turkmenistan, Uzbekistan, Libya, Samoa, South Africa, Gabon, Egypt, Marshall Islands, Viet Nam, Iraq, Morocco, Kyrgyzstan, Mozambique, Tjikistan, Cabo Verde.
Component1	Brazil, Bangladesh, Colombia, Thailand, Philippines, Guyana, Indonesia, Uruguay, Dominica, Palau, Brunei Darussalam, Kribati, Botswanna, Bahrain, Fiji, Central Africa Republic, United Arab Emirates, Honduras, Azerbaijan, Dominican Republic, Palestine, Moldova, Saint Vincent and the Grenadines, Grenada, Saint Lucia, Paraguay, Ecuador, Ukraine, El Salvador, United States, Cyprus, Bosnia and Herzegovina, Spain, Oman, Italy, Korea, China.
Component 2	Congo, Ghana, Belarus, Rwanda, Niger, Togo, Barbados, Senegal, Sudan, Mali, Nepal, Ethiopia, Costa Rica, Belgium, Germany, Hungary, New Zealand, Russian Federation, Estonia, Switzerland, Montenegro, Slovakia, Finland, Slovenia, Malta, Latvia, Yemen, North Macedonia, Algeria, Sweden, Albani, Czechia, Canada.

It is important to mention that Figure 3 presents the connections that the nodes belonging to each set have in the five layers.

Based on the information presented in Figure 3 and Table 3, we can see that in Component 1, there are countries as Brazil, China, Italy (among others) with a high human development index; however, these countries present high percentage in contagion and deaths. On the other hand, in Component 2, there are countries as Congo, Niger, Montenegro (among others) with a low percentage of contagion, and low human development index; therefore, the characteristic that both types of countries share is the low number of air travels.

For the separator set, there are countries with different values of characteristics (for more information, see the URLs presented in Annex) according to the percentage of the population who infect or died by day, an average of the projected real GDP, average consumer prices, human development index, the average of the Corruption perception index, average current health expenditure (% of GDP), average population and number of air travels.

For example, in the socioeconomics and population characteristics: Norway, Ireland, Hong Kong, Sweden (among others) have high values in Human development index (HDI), life expectancy at birth, expected years of schooling, percentage of infected by day and the rate of deaths by day; while Iran, Mauritius, Panama, Georgia, Sri Lanka (among others) have average values and Viet Nam, Iraq, Morocco, Kyrgyzstan, Tajikistan, Cabo Verde (among others) have low values.

Based on the information about the number of infections and deaths, we can see that some countries as the United States, Italy, and China, which are considered the countries with the most diseases and deaths, are not in the set of the most spreaders. On the other hand, some countries that are in the spreaders set are: Norway, Ireland, and Denmark that present a high percentage of contagion; Mexico, Venezuela, and Egypt with an average percentage and countries like India, Nicaragua, and Mozambique have a low percentage.

**FIGURE 4.**
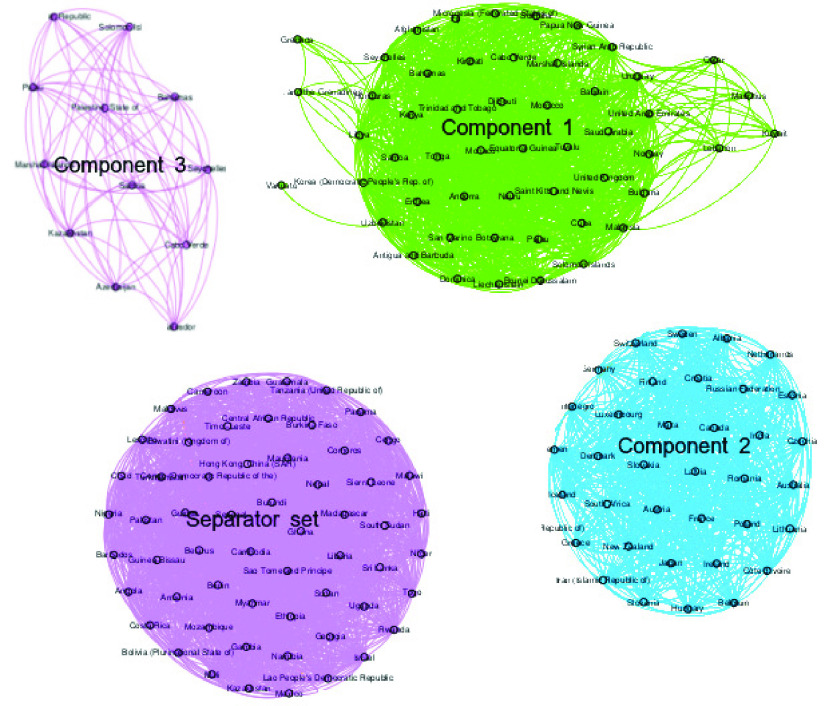
Graph of the sets of countries after the rupture of the MCGC (GDP network).

Now, to analyze the relationship between the GDP and the spread of COVID-19, we present Figure 4 and Table 4, which perform the sets of countries after the rupture of the MCGC in GDP and health network.

**TABLE 4:** Sets of Countries After the Rupture of the MCGC (GDP and Health Network)

GDP and health network	
Separator set	Norway, Switzerland, Ireland, Germany, Hong Kong, Iceland, Sweden, Finland, New Zealand, United States, Belgium, Liechtenstein, Austria, Luxembourg, Israel, Korea, Spain, Malta, Italy, Cyprus, Greece, Poland, Lithuania, United Arab Emirates, Saudi Arabia, Slovakia, Bahrain, Croatia, Oman, Russian Federation, Belarus, Kazahstan, Bulgaria, Kuwait, Bahamas, Seychelles, Serbia, Trinidad and Tobago, Mauritius, Albania, Costa Rica, Sri Lanka, Saint Kitts and Nevis, Antigua and Barbuda, Bosnia and Herzegovina, Mexico, Colombia, Armenia, Algerian, North Macedonia, China, Ecuador, Azerbaijan, Ukraine, Dominician Republic, Tunisia, Mongolia, Lebanon, Botswana, Jamaica, Venezuela, Dominica, Jordan, Tonga, Philippines, Turkmenistan, Uzbekistan, ybia, Samoa, Marshall Islands, Palestine, Guyana, Tajikistan, Cabo Verde, Namibia, Timor-Leste, Honduras, Bhutan, Bangladesh, Micronesia, Sao Tome and Principe, Lao People’s Democratic Republic, Vanuatu, Cambodia, Kenya, Nepal, Pakistan, Nigeria, Benin, Lesotho, Cote d’Ivoire, Togo, Haiti, Afghanistan, Dijbouti, Gambia, Guinea, Liberia, Mozambique, Sierra Leone, Burkina Faso, Eritrea, Mali, Burundi, Central African Republic, Niger, Monaco, Nauru, San Marino, Somalia, Tuvalu.
Component1	Moldova, Nicaragua, El Salvador, Palau, Brunei, Darussalam, Fiji, Malaysia, Cuba, Nigeria, Papua New Guinea, Tonga, United Kingdom, Uruguay, Ecuador, Equatorial Guinea, Qatar, Democratic People’s Rep. of Korea, Syrian Arab Republic, Congo, Morocco, Denmark, Canada, Montenegro, Iran, Hungary, Japan, India, Romania, Czechia, Slovenia, France, Netherlands, Yemen,

It is important to mention that Figure 4 presents the connections that the nodes belonging to each set have in the 5 layers.

As in the previous case, based on the information presented in Figure 4 and Table 4, we can see that in each set (separator, component 1 and component 2), there are countries with different values in the characteristics.

For example, in components 2 and 3, there are countries such as Ghana, Nepal, Uganda, Egypt (among others) with low human development index, and the high percentage of contagion and deaths.

For the socioeconomics, GDP and health characteristics, in the separator set (spreaders), are countries as Norway, Switzerland, Germany (among others) that have high values in Human development index (HDI), life expectancy at birth, expected years of schooling, percentage of infected and deaths by day; countries as Costa Rica, Mexico, Colombia, Armenia (among others) that have average values and countries like Nauru, San Marino, Somalia, Tuvalu (among others) have low values.

Based in the information about the flow in air travels, we can affirm that the countries in the separator set for both infection and GDP networks have an increase in the rate of contagion since in addition to being those countries where there is a higher flow in air travels (inbound and outbound), economies are based on business and transactions with other countries (which are in the same situation).

Finally, based on the results shown in Table 3 and Table 4, we can see that in order to mitigate a second outbreak of COVID-19 in the world, the countries that are in the union of both separator sets must reinforce their sanitary measures (in the area). On the other hand, the countries that are at the intersection of the two separator sets, in addition to strengthening their sanitary measures, must regulate the airflow in order to contain the spread of the disease.

## V. Limitations of the Study and Discussion

The analysis developed in this work, is based on the modeling of two multiplex networks, which consist of 5 layers; where each layer shows the similarities of the countries for the different types of characteristics.

The idea of model five different types of layers is to be able to obtain an identification of the spreader nodes, based on a multi-criteria analysis that reduces the disadvantages of using only one set of nodes; thus, the technique is capable of identifying those countries that, based on different characteristics, are the most spreaders of COVID-19.

We apply the multiplex network approach using the adaptation of the Vertex Separator Problem, in order to identify those countries that, when their links are eliminated from all the layers of the network, cause the rupture of the system and, therefore, contain the spreaders countries of COVID-19.

The main advantage of this approach is that it allows information from various fields to be combined, such as economic, health, and transportation. Thus, with this approach, we can quantify the relationship between the different countries and model the networks that help to understand the dynamics of the system to be analyzed (in this case, the spread of COVID-19).

It is important to mention that, in this work, we are quantifying the dynamics of behavior of COVID-19 with the information available until May 15, 2020.

Therefore, a limitation of the study is that the drastic change of one or more variables causes the dynamics of the system to change. Then, because the information about COVID-19 is updated every day, the set of spreader countries that we find can be maintained if the behavior of the variables is maintained; otherwise, we must re-model the networks to carry out a new analysis.

Although we are schematizing the dynamics of the spread behavior of COVID-19, the analysis is susceptible to the existence of those elements that drastically alter one or more layers of the entire multiplex network. However, as long as the behavioral trends are maintained (values within the 95% level of certainty show in Table 1), the set of spreader countries found, will not change.

## VI. Conclusions

In this work, we present an analysis of the countries that are spreaders of COVID-19, based on the main socio-cultural, economic, and connection characteristics, such as GDP, life expectancy, number of air travel, and budget for health, among others.

The results show that the methodology, can cause the rupture of the 5-layer multiplex network and help identify the spreader countries and obtains a classification of the countries based on their characteristics, where, in the spreaders set, the countries have high, medium or low values in the different socio-cultural and economic aspects; however, the characteristic that everyone shares are the high value in air connections.

On the other hand, we can affirm that to mitigate a second outbreak of COVID-19 in the world, the countries that are in the union of both separator sets must reinforce their sanitary measures; in contrast, the countries that are at the intersection of the two separate sets, in addition to improving their sanitary measures, must regulate airflow to contain the spread of the disease.

Based on the information collected and modeled (until May 15, 2020), we can affirm that, by changing the relationships of the air flow, the risk of a second outbreak of COVID 19 can be minimized; however, we cannot quantify how much it can help.

Finally, in order to study and analyze the behavior of the propagation of COVID-19, we are currently developing a simulation system, which is based on prediction models of COVID-19 in 165 countries, and the approach presented in this work.

Annex

Now, we present the URLs for the information used to model the six networks used as layers for the two 5-layer multiplex networks.

• Information about Human Development Index: https://drive.google.com/file/d/136y0449h4sfM86Ye-qeOrL8x kx9ixFAE/view?usp=sharing
• Information about deaths and infections: https://drive.google.com/file/d/1PqwJB-F3Y9WWFhVRwBUDydO CkqvT7ECn/view?usp=sharing
• Information about air travels: https://drive.google.com/file/d/18PA8JMnzxueXLU1MzjkEya3htBe4hrBw/ view?usp=sharing
• Information about GDP: https://drive.google.com/file/d/1HhAEpN-WzEBJXAzJeNbF5iSNm8S2xi1Q/view? usp=sharing
